# Sox15 Methylation Inhibits Cell Proliferation Through Wnt Signaling in Hepatocellular Carcinoma

**DOI:** 10.3389/fonc.2022.842312

**Published:** 2022-03-22

**Authors:** Bajin Wei, Hao Chen, Xiaobin Chen, Danjing Guo, Liangjie Hong, Shusen Zheng

**Affiliations:** ^1^ Division of Hepatobiliary and Pancreatic Surgery, Department of Surgery, The First Affiliated Hospital, School of Medicine, Zhejiang University, Hangzhou, China; ^2^ National Health Commission (NHC) Key Laboratory of Combined Multi-organ Transplantation, The First Affiliated Hospital, School of Medicine, Zhejiang University, Hangzhou, China; ^3^ Key Laboratory of the Diagnosis and Treatment of Organ Transplantation, Research Unit of Collaborative Diagnosis and Treatment for Hepatobiliary and Pancreatic Cancer, Chinese Academy of Medical Sciences (2019RU019), The First Affiliated Hospital, School of Medicine, Zhejiang University, Hangzhou, China; ^4^ Key Laboratory of Organ Transplantation, Research Center for Diagnosis and Treatment of Hepatobiliary Diseases, The First Affiliated Hospital, School of Medicine, Zhejiang University, Hangzhou, China; ^5^ Department of Lung Transplantation and General Thoracic Surgery, The First Affiliated Hospital, School of Medicine, Zhejiang University, Hangzhou, China; ^6^ Key Laboratory of Pulsed Power Translational Medicine of Zhejiang Province, Hangzhou, China

**Keywords:** SOX15, HCC, Wnt signaling, cell proliferation, methylation

## Abstract

The expression of the SRY-Box Transcription Factor 15 (Sox15) is reduced by DNA methylation, and its progression is suppressed within numerous tumors. However, its effect on hepatocellular carcinoma (HCC) remains unknown. In the present work, the clinical importance and function of Sox15, as well as the underlying molecular mechanism, were explored within HCC. The expression of Sox15 is reduced and positively correlated with prognosis in HCC as analyzed by GEPIA (Gene Expression Profiling Interactive Analysis) and OncoLnc. Meanwhile, the hypermethylated Sox15 promoter CpG-site predicted a dismal HCC prognosis. Besides, ectopic Sox15 expression within the HCC cells (LM3, HUH7, SK-hep-1) remarkably inhibited *in vitro* cell growth and inhibited xenograft tumorigenesis in the nude mice. Moreover, Sox15 inactivated the Wnt pathway under both *in vivo* and *in vitro* conditions. To summarize, Sox15 played a tumor suppressor role within the HCC *via* the inactivated Wnt pathway. Sox15 and CpG-site methylation of its promoter are the factors that independently predict the prognosis of HCC.

## Introduction

The global cancer-associated mortality showed an increasing trend before 1991; the trend decreased till 2017 due to the long-term reduction in the mortality of the four major tumor types (lung cancer (LC), colorectal (cancer CRC), prostate cancer (PCa), and breast cancer (BC)) (1). Nonetheless, the long-term mortality of liver cancer rapidly increases in women and stabilizes in men. For hepatocellular carcinoma (HCC) of diverse pathogenic mechanisms, the average age of onset and morbidity has elevated over the last three decades (2). Currently, HCC has become the second most common cause of cancer-associated global mortality and poses a great burden on public health (3). HCC, which accounts for around 85-90% of all primary liver cancers, shows a particularly high occurrence in China due to the high prevalence of chronic hepatitis B (CHB) infection (4). HCC can be effectively controlled by the large-scale implementation of validated interventional measures like vaccination (5). Moreover, there have been improvements in therapies for HCC treatment such as hepatic resection, liver transplantation, transarterial chemoembolization (TACE), and small molecule targeted drugs (6). However, the urgency to explore more efficient therapies for HCC persists.

Various genetic changes like structural rearrangements, nucleotide substitutions, epigenetic alterations, and viral genome integration are involved in cancer occurrence (7). Among them, epigenetic modifications like abnormal DNA methylation remodel gene expression profiles within the malignancies (8). DNA methylation is an epigenetic phenomenon that has received extensive investigation. Disturbances in methylation patterns may alter gene transcription and thereby significantly affect the cancer biological behaviors (9). Because of the reversibility, DNA methylation together with the resultant RNA methylation facilitates the development of new markers and treatments against HCC (10). In HCC, the hypermethylated genes are remarkably enriched into the CpG islands (CGIs). By combining integrative promoter arrays with methylated DNA immunoprecipitation/hybridization, about 3,700 hypomethylated promoters have been detected within the HCC samples (11). Cancer can be detected in a non-invasive manner as early as four years before the detection of DNA methylation in the tumor by the non-invasive blood test (12). The methylated genes are mainly involved in cell growth, invasion, migration, and signaling, and hence, play important roles in cancer development.

The Sry-related high-mobility-group (HMG) box genes (Sox) belong to the transcription factor (TF) family, which has important functions in determining cell fate in the process of development (13). In mammals, Sox15 is the only member in group G of Sox (14). Sox15 exerts an important effect on deciding myogenic cells in the early development of skeletal muscle (13) and overexpresses in the ectopic endometrium of endometriosis patients (15). Certain SOX family members may exert effects on regulating cell proliferation. Sox15 expression is reduced by DNA methylation in many cancers like pancreatic (16), endometrial (17), colorectal cancers (18), and glioma (19). Upregulation of Sox15 could repress cancer development by suppressing cell growth, invasion, and migration or by promoting cell apoptosis. Based on the above results, Sox15 can be considered a potential tumor suppressor gene (TSG). Sox15 shows low expression within HCC, which indicates a poor prognosis. In this research, the functions and underlying mechanism of Sox15 in HCC were explored.

## Materials and Methods

### Tissue Samples

Tumor and paired, non-carcinoma samples were collected from 144 HCC patients of the First Affiliated Hospital of Medical School of Zhejiang University during 2008-2015. Clinicopathological characteristics of each case were recorded during the follow-up visits every six months. The samples were preserved in a deep freezer at -80°C or formalin-fixed and paraffin-embedded for further research.

### HCC Cells

The HCC cell lines (SK-hep1, Huh7, LM3, SNU-449, and LO2) required for the study were procured from the Shanghai Cell Bank, Chinese Academy of Sciences. The cells were cultivated in the alpha-MEM (BI industry, Israel) medium containing 10% fetal bovine serum (FBS, Gemini Bio, USA).

### Demethylation Treatment

Cells (1 × 10^6^/mL) were inoculated overnight into the 6-well plates. Subsequently, they were treated with 5-Azacytidine (10 µM, Sigma, USA) for 96 h followed by treatment with 300 mM TSA (tryptic soy agar) (Sigma, USA) for 24 h. The cells were then harvested for further DNA and RNA measurements.

### RNA Extraction and Quantitative Real-Time RT-PCR (qRT-PCR)

RNA was extracted, and qRT-PCR was conducted according to a previous report (20). GAPDH served as the internal control. Primers used in this work are listed below:

Sox15 forward: 5’-CAAGATGCACAACTCCGAGA-3’
Sox15 reverse: 5’-GGAGCCTAGGGT CACTCTGA-3’
GAPDH forward: 5’-AATGGGCAGCCGTTAGGAAA-3’
GAPDH reverse: 5’-GCGCCCAATACGACCAAATC-3’.

### Protein Extraction and Western Blotting (WB) Assay

Proteins were extracted, and the WB assay was conducted according to a previous report (20). GAPDH was the endogenous reference. The following primary antibodies were utilized in this work. Sox15 (HPA067196) was purchased from Sigma (USA). β-catenin (8480S), CD44 (3570T), and MET (8198T) were purchased from CST.

### Genomic DNA (gDNA) Bisulfite Modification as Well as Promoter Methylation Analysis

gDNA bisulfite modification was performed using a specific kit (EpiTect Bisulfite Kits, Qiagen, USA). Next, through 40 PCR cycles, mDNA was amplified using the synthesized MSP (methylation specific PCR) or USP (unmethylation specific PCR) primers. Later, the PCR products were measured by DNA electrophoresis. The primer sequences are as given below:

Sox15 MSP primer forward: 5’-TGAGAGTTAAGGTAGGTTGTTTAATTTG-3’
Sox15 MSP primer reverse: 5’-ACAAATTTTAACTTCAATCATC-3’
Sox15 USP primer forward: 5’-GTTAAGGTAGGTTGTTTAATTCGA-3’
Sox15 USP primer reverse: 5’-ATAACAAATTTTAACTTCAATCGTC-3’.

### Overexpression of Sox15 and Lentivirus and Cell Transfection

Sox15 overexpression vectors and lentiviruses were procured from GeneCopoeia. The Sox15 overexpression vector or lentivirus was transfected into the Huh7, SK-hep-1, and LM3 cells using polybrene (8 µg/mL, Sigma). The overexpression efficiency was tested 72 h post-infection.

### Cell Viability Test

Cells (1 × 10^5^/mL) were inoculated into 96-well plates. The CCK-8 assay was performed at 24 h, 48 h, 72 h, and 96 h, according to specific protocols. Finally, the ELx800 Absorbance Microplate Reader (BioTek) was utilized to measure the colored solution at the wavelength of 450 nm.

### Cell Colony Assay

Cells (1 × 10^3^/mL) were cultured into 6-well plates for two weeks. Giemsa staining was performed in line with specific protocols. Image J was adopted for result picturing and analysis.

### HE and Immunohistochemistry (IHC)

Immunohistochemistry was carried out according to a previous description (20). The primary antibodies utilized in the present work included Sox15 (HPA067196, Sigma) and Ki67 (9499T, CST).

### *In Vivo* Xenograft Model

The animal study protocols were approved by the Animal Ethics Committee of the Zhejiang University. Briefly, mice were given a subcutaneous injection of PBS (0.1 ml) that contained the expressing control vector or Sox15 LM3 cells (1 × 10^6^) in the flask, respectively. After around two weeks, the tumor size was detected using the digital caliper. The formula of V = ½ L×W^2^ was adopted to calculate the tumor volumes. Here, ‘L’ stands for the length (long diameter), whereas ‘W’ represents the width (short diameter).

### Statistical Analysis

The results were displayed in the form of mean ± SD. SPSS16.0 (IBM Corporation, New York, USA) was employed for statistical analysis.

## Results

### The Expression of Sox15 Was Reduced and Positively Correlated With Prognosis in HCC

**Figure 1:**
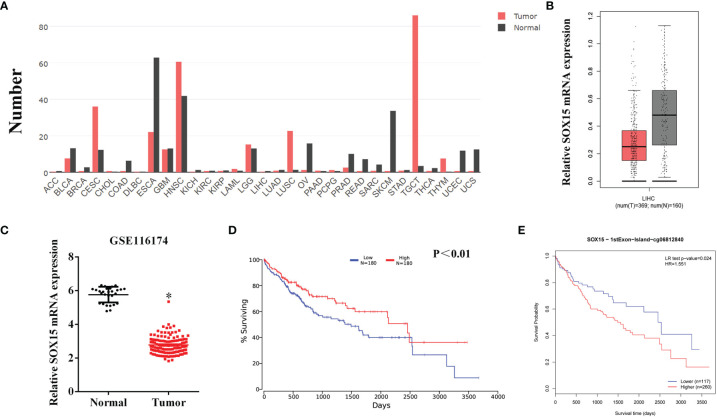
The expression of Sox15 is reduced and positively correlates with prognosis in HCC as analyzed by TCGA and GEO data. **(A)** Expression pattern of Sox15 in a pan-cancer perspective. **(B)** Sox15 mRNA levels within the HCC and normal tissues were detected by the TCGA database. **(C)** Sox15 mRNA expression within HCC and matched, non-carcinoma samples analyzed by GSE116174 data. **(D)** KM analysis suggested that HCC cases with decreased Sox15 levels suffer a worse OS. p<0.01. **(E)** KM analysis suggested that HCC cases showing increased Sox15 promoter methylation suffer a worse OS. *p < 0.05.

**Figure 2:**
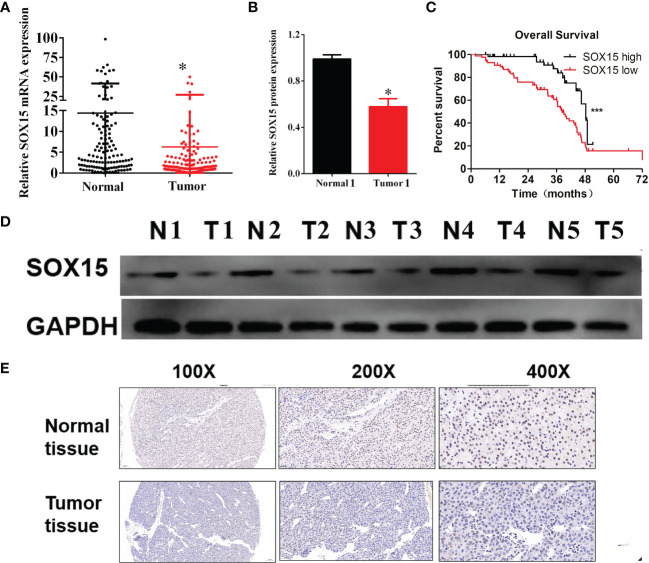
The expression of Sox15 is reduced and positively correlates with prognosis in HCC as analyzed by our cohort. **(A)** Sox15 mRNA levels within HCC as well as paraneoplasia samples measured through RT-qPCR. **(B)** Quantification of Sox15 protein levels of the representative HCC and its paraneoplasia samples shown in panel **(C, D)** KM analysis suggested that HCC cases with reduced Sox15 levels suffered worse OS. **(D)** Sox15 protein levels of the indicated HCC and its paraneoplasia samples measured by Western Blot. **(E)** Sox15 protein levels within HCC and its paraneoplasia tissues measured by IHC. *p < 0.05, ***p < 0.001.

The expression of Sox15 was examined by GEPIA in a variety of cancers. The expression was reduced in most cancer types (**Figure 1A**) and also in HCC (**Figure 1B**). The higher expression of Sox15 was confirmed from the GSE116174 data (**Figure 1C**). Moreover, the correlation between Sox15 and the prognosis of the HCC patients was analyzed by OncoLnc. The Sox15 expression level showed a positive correlation with prognosis (**Figure 1D**). Further, the relationship between Sox15 promoter hypermethylation and the patient prognosis was analyzed by TCGA data, wherein hypermethylation of Sox15 was correlated to poorer patient survival (**Figure 1E**). Cancer and paired non-carcinoma samples from 144 HCC cases were collected to confirm the results from bioinformatics. The Sox15 mRNA and protein expression was detected through RT-PCR WB and IHC assays, respectively. The Sox15 protein and mRNA expression levels were downregulated in the HCC samples (**Figures 2A, B, D**). Based on the IHC analysis, Sox15 showed a nuclear localization (**Figure 2E**). Further, the association between the Sox15 level and patient survival was determined from the mRNA data. According to the findings, a reduced Sox15 level predicted poorer patient survival and was associated with the tumor size (**Figure 2C** and **Table 1**). Taken together, these findings suggested that a reduced Sox15 level predicted poor survival and a higher malignant grade of HCC.

**Table 1:** Relationship between Sox15 expression and clinicopathological feature in 144 HCC cases.

Characteristic	Sox15 expression level		P-Value
	High	Low	
Mean age	58.8 ± 11.1	55.5 ± 10.3	0.073
AFP			0.906
<400	43	63	
≥400	15	23	
Tumor grade			0.220
Low and moderate	37	46	
High	21	40	
Size (cm)			0.001
<5	36	29	
≥5	22	57	
Tumor number			0.139
1	48	62	
≥2	10	24	
PVTT			0.326
Absent	44	71	
Present	14	15	

AFP, alpha-fetoprotein; PVTT, portal vein tumor thrombus.

### The Promoter of Sox15 Is Hypermethylated and Correlated With Poorer Prognosis in HCC

**Figure 3:**
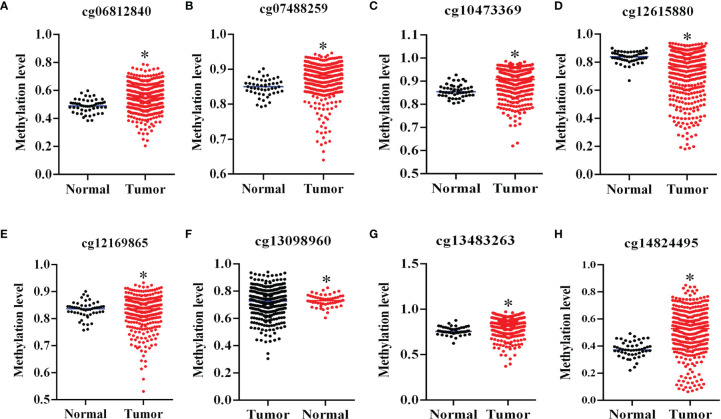
Sox15 silencing was governed by promoter methylation in HCC. **(A–H)** Sox15 promoter methylation level in CpG sites in HCC detected through TCGA data. *p < 0.05.

**Figure 4:**
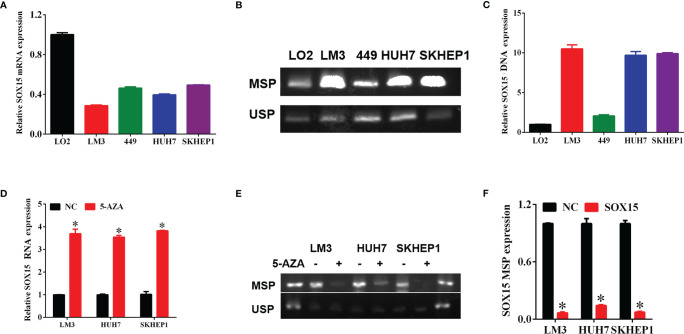
The silencing of Sox15 was governed through promoter methylation within HCC cells. **(A)** Sox15 mRNA levels within HCC cells measured through RT-qPCR. **(B, C)** Sox15 promoter methylation status is measured by MSP and USP and quantification. **(D)** Sox15 mRNA levels within HCC cells measured by RT-qPCR post-exposure to 5-Aza. **(E, F)** Sox15 promoter methylation status is measured by MSP and USP and quantification post-exposure to 5-Aza. MSP, methylation specific PCR; USP, unmethylation specific PCR; *p < 0.05.

The expression analysis of the Sox15 promoter methylation indicated that the CpG sites in the Sox15 promoter region were hypermethylated in the tumor samples (**Figure 3**) and HCC cell lines (**Figures 4B, C**). However, in normal samples, the methylation was low, and the Sox15 levels conformed to those within the HCC cells (**Figure 4A**). For further validation, the HCC cells with hypermethylated Sox15 (LM3, HUH7, and SK-hep-1) were exposed to 5-Azacytidine (methyltransferase inhibitor), and the levels of Sox15 promoter methylation and Sox15 mRNA were measured. The results indicated that methyltransferase inhibitors demethylated the Sox15 promoter and restored the expression of the Sox15 mRNA (**Figures 4D–F**). Thus, the Sox15 promoter DNA hypermethylation was related to transcriptional silencing within HCC and negatively correlated with prognosis in HCC.

### Sox15 Suppresses the *In Vitro* Proliferation of the HCC Cells

**Figure 5:**
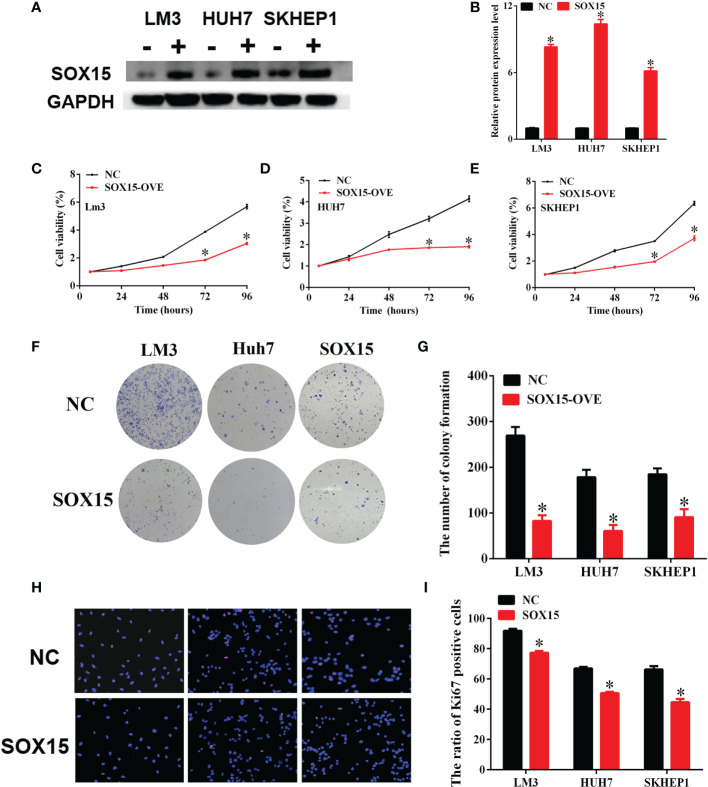
Sox15 inhibits the *in vitro* growth of HCC cells. **(A, B)** Sox15 overexpression efficiency in HCC cell lines detected through WB assay and quantification. **(C–E)** Role of Sox15 overexpression in HCC cell viability measured by CCK-8 assay. **(F, G)** Role of Sox15 overexpression in the clone-forming capacity of HCC cells and quantification. **(H, I)** Role of Sox15 overexpression in HCC cell viability measured by Ki67 IHC and quantification. *p < 0.05.

HCC cells with hypermethylated Sox15 levels (LM3, HUH7, and SK-hep-1) were further chosen to investigate HCC cell growth. Sox15 was overexpressed in these three HCC cell lines (**Figures 5A, B**), and clone forming and CCK-8 assays were performed. The overexpression of Sox15 significantly inhibited cell viability (**Figures 5C–E**) as well as colony formation (**Figures 5F, G**). IHC of Ki67 was also performed to investigate the effect of Sox15 on cell proliferation. Sox15 overexpression remarkably decreased the Ki67 protein level within the nucleus (**Figures 5H, I**). The application of methyltransferase inhibitors to these three cell lines significantly inhibited cell viability.

### Sox15 Inhibits *In Vivo* HCC Carcinogenesis

**Figure 6:**
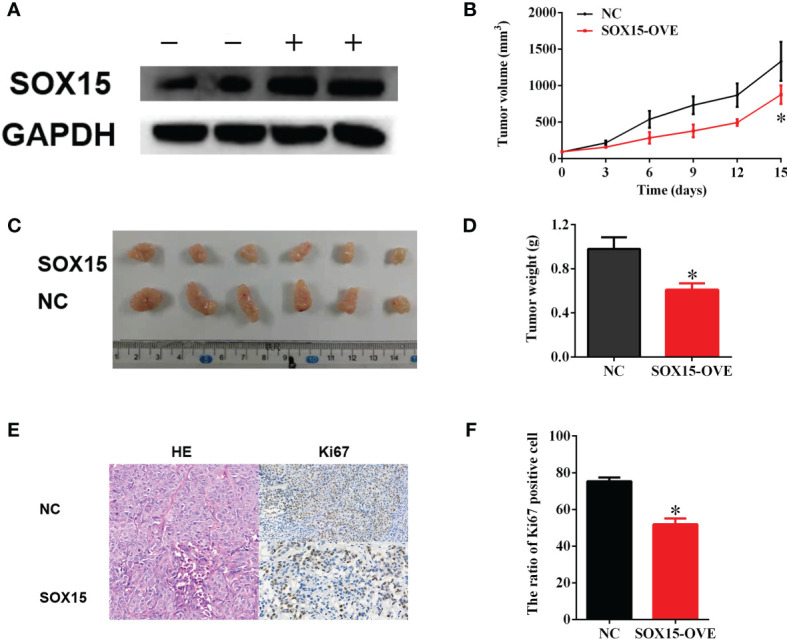
Sox15 inhibits *in vivo* HCC tumorigenicity. **(A)** The overexpression efficiency of Sox15 within the HCC cells as measured through the WB assay. **(B)** The tumor growth curve in nude mice. **(C, D)** The typical image and tumor weight of Sox15-transfected and vector-transfected tumors at the time of sacrifice. **(E, F)** Classic HE and Ki67 IHC of tumor samples and quantification. *p < 0.05.

For identifying the above findings under *in vivo* conditions, a nude mice model was constructed by subcutaneously injecting LM3-Sox15 or LM3-vector cells into the mice. Before injection, the successful transfection of the LM3 cells was confirmed by western blot (**Figure 6A**). The mice were observed every three days and sacrificed when the diameter of the tumor reached 1 cm. The growth curve of nude mice is presented in **Figure 6B**. The typical image of Sox15-transfected and vector-transfected nude mice at the time of sacrifice is shown (**Figure 6C**). Tumor weight was significantly smaller in the Sox15-transfected mice (**Figure 6D**) compared to that in the vector-transfected mice. Further, the tumor samples from the nude mice were detected through IHC and HE staining. Sox15 transfection inhibited the expression of Ki67 and proliferation (**Figures 6E, F**). Based on the above results, Sox15 acted as a TSG (tumor suppressor gene) during HCC tumorigenicity under *in vivo* conditions.

### Sox15 Inactivated the Wnt Pathway in HCC

**Figure 7:**
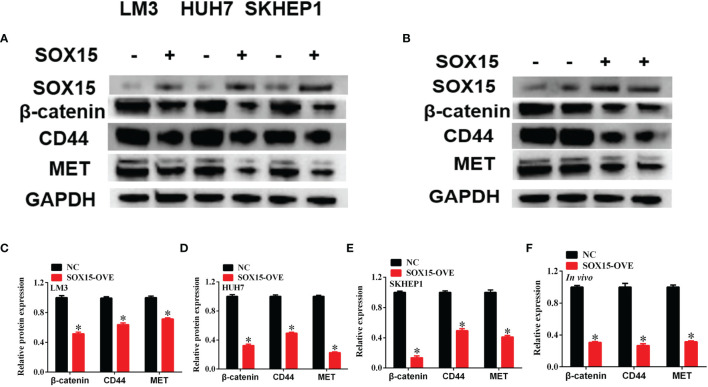
Sox15 inactivates Wnt pathway in HCC. **(A–F)** The key members of Wnt pathway were measured by western blot after Sox15 overexpression under *in vitro* **(A, C–E)** and *in vivo* **(B, F)** conditions. *p < 0.05.

Sox15 is reported to perform its function on cell behavior *via* the Wnt pathway. The expression of Wnt pathway members was measured under *in vivo* and *in vitro* conditions by the WB assay. β-catenin, CD44 and MET levels remarkably decreased, following Sox15 overexpression under both *in vivo* and *in vitro* conditions (**Figures 7A–F**). These findings suggested that Sox15 played its role *via* the Wnt pathway.

## Discussion

In this study, the transcription factor Sox15 was identified as an inactivated tumor suppressor gene (TSG) *via* promoter methylation. Based on such findings, Sox15 mRNA level decreased within varieties of cancer, consistent with studies (16–19). In HCC, the expression of the Sox15 mRNA was downregulated, and reduced Sox15 expression was related to a poorer prognosis. In paired, primary HCC samples from patients, both Sox15 mRNA and protein levels decreased within the tumor tissue; a lower expression of Sox15 was related to bigger tumor size and poorer prognosis. Data from BGS (bisulfide genome sequencing) presented that the CpG sites in the Sox15 promoter region were hypermethylated both in HCC cells and in tissues. Sox15 downregulation was subsequently restored by treating with DNA methyltransferase inhibitors. Moreover, the methylation status of Sox15 was a factor that independently predicted OS for liver cancer cases. Promoter methylation for prognosis prediction is reported within diverse cancer types. In gall bladder cancer, determining DNA methylation patterns provides the possible diagnostic, prognostic markers, and/or therapeutic targets (21). In leukemia, methylation of MRD (minimal residual disease) may be used as a biomarker to study patient relapse (22). Also, DNA methylation of PD-L2 can estimate the progression-free survival (PFS) for cases receiving anti-PD-1 treatment in melanoma (23). Therefore, these results suggest that Sox15 and the methylation of its promoter may be the clinical prognostic biomarkers for HCC.

As suggested by various *in vivo* and *in vitro* functional experiments, Sox15 functions as the TSG in HCC. Undoubtedly, the immortalized proliferation of tumor cells is an important feature (24). Ectopic Sox15 expression within the HCC cells (LM3, Huh7, and SK-hep-1) suppressed the cell growth, as evidenced through clone forming and CCK-8 assays. Meanwhile, 5-AZA administration also inhibited cell proliferation in these three cell lines as detected by the CCK-8 assay. Ki67 serves as the classic marker to predict cell growth (25), and it was selected to detect cell proliferation by IHC. Under *in vitro* conditions, Sox15 overexpression significantly suppressed the nuclear Ki67 expression. In addition, under *in vivo* conditions, Sox15 overexpression significantly inhibited the tumor growth as measured by tumor size and weight. Moreover, the expression of Ki67 was also downregulated by Sox15 overexpression. Collectively, Sox15 was identified as the TSG in HCC in this study for the first time.

The wnt/β-catenin pathway, which shows a high conservation degree, regulates embryonic development, cellular proliferation, and differentiation (26). It plays an important function in regulating embryogenesis, such as the development, zonation, as well as maturation of the liver and gall bladder. Under normal conditions, this pathway is inactivated within the healthy mature liver, but it may be re-activated in the process of cell renewal and regeneration (27). An increasing number of studies have suggested that the abnormally activated WNT/β-catenin pathway plays a key role in initiating and developing HCC (28). Meanwhile, Sox15 has been discovered to be the new TSG within esophageal and pancreatic cancers, and it may regulate the WNT/β-catenin pathway (29). Results from *in vivo* and *in vitro* experiments indicated that overexpression of Sox15 inhibits the Wnt/β-catenin pathway activation. Based on the above findings, it was concluded that Sox15 plays its role *via* the Wnt/β-catenin pathway. Of note, the SOX15 gene transcriptional silencing *via* promoter methylation in HCC cells can also be linked to copy number alterations, as well as miRNA molecules may play a key role in the epigenetic regulation of SOX15.

To summarize, Sox15, together with its methylation status, is the independent prognostic factor for overall survival in HCC. Additionally, Sox15 is a novel TSG that inhibits cell proliferation in HCC. The tumor-suppressive function of Sox15 is mediated by downregulating the Wnt pathway and its target genes.

## Data Availability Statement

The datasets presented in this study can be found in online repositories. The names of the repository/repositories and accession number(s) can be found in the article/**Supplementary Material**.

## Ethics Statement

The studies involving human participants were reviewed and approved by Clinical Research Ethical Committee of the First Affiliated Hospital, Zhejiang University School of Medicine. The patients/participants provided their written informed consent to participate in this study. The animal study was reviewed and approved by The Lab of Animal Experimental Ethical Inspection of the First Affiliated Hospital, Zhejiang University School of Medicine.

## Author Contributions

Conceptualization, BW and SZ. Methodology, HC, XC, and DG. Resources, LH. Writing—original draft preparation, BW and HC. All authors have read and agreed to the published version of the manuscript.

## Funding

This research was supported by Innovative Research Groups of National Natural Science Foundation of China (No. 81721091), National S&T Major Project (No. 2017ZX10203205), Zhejiang International Science and Technology Cooperation Project (NO.2016C04003), Research Unit Project of Chinese Academy of Medical Sciences (2019-I2M-5-030) and Grant from Health Commission of Zhejiang Province (JBZX-202004).

## Conflict of Interest

The authors declare that the research was conducted in the absence of any commercial or financial relationships that could be construed as a potential conflict of interest.

## Publisher’s Note

All claims expressed in this article are solely those of the authors and do not necessarily represent those of their affiliated organizations, or those of the publisher, the editors and the reviewers. Any product that may be evaluated in this article, or claim that may be made by its manufacturer, is not guaranteed or endorsed by the publisher.
