# Supplementary material for: Sox15 Methylation Inhibits Cell Proliferation Through Wnt Signaling in Hepatocellular Carcinoma
Source: Front Oncol. 2022 Mar 22;12:842312. doi: 10.3389/fonc.2022.842312 (PMC8980349; doi:10.3389/fonc.2022.842312)
Supplement: Supplementary file 1 [file DataSheet_1.docx]

1. The expression of SOX15 mRNA in liver cancer and normal tissues

| Tumor | Normal | Tumor | Normal | Tumor | Normal |
| --- | --- | --- | --- | --- | --- |
| 1.002293 | 14.461260 | 5.955166 | 6.030939 | 0.6571325 | 2.708838 |
| 3.569403 | 122.469800 | 3.284063 | 62.300780 | 0.1104663 | 9.701175 |
| 3.159193 | 57.371090 | 15.498870 | 58.755010 | 1.446094 | 1.822388 |
| 9.205393 | 21.155650 | 50.078260 | 65.497870 | 1.374363 | 0.6598115 |
| 0.593507 | 57.878650 | 14.621120 | 17.381930 | 0.1883355 | 14.354020 |
| 0.8273934 | 4.121251 | 5.915200 | 25.625800 | 0.6175964 | 2.760581 |
| 4.677772 | 15.455240 | 2.576910 | 16.112230 | 5.439838 | 0.9086362 |
| 0.5539362 | 6.699557 | 3.057582 | 16.000000 | 0.6478815 | 7.098333 |
| 1.054202 | 3.643803 | 1.002874 | 10.115670 | 0.8464859 | 3.869798 |
| 2.709086 | 44.263420 | 0.3312809 | 2.543447 | 6.362661 | 13.557530 |
| 0.782470 | 98.307880 | 0.8878184 | 3.097087 | 1.984352 | 2.840104 |
| 0.8321992 | 4.401949 | 0.9630177 | 5.790929 | 0.1988521 | 4.229322 |
| 0.6963475 | 3.091268 | 1.141503 | 1.928818 | 1.624252 | 8.340289 |
| 1.458168 | 5.407268 | 1.080080 | 2.672031 | 3.693540 | 3.511746 |
| 1.370702 | 14.095910 | 0.2787605 | 1.602613 | 7.069053 | 3.357152 |
| 0.9921182 | 126.245700 | 0.1922909 | 1.656747 | 4.425180 | 5.508708 |
| 3.739038 | 36.636380 | 0.7820067 | 2.536467 | 1.016659 | 1.204582 |
| 1.446651 | 6.358208 | 0.9822319 | 8.105332 | 1.657029 | 7.653659 |
| 1.495027 | 7.052476 | 0.1880248 | 1.988717 | 1.547788 | 0.04720416 |
| 9.688921 | 26.943490 | 0.4403265 | 1.896252 | 0.7128544 | 11.941570 |
| 7.439017 | 17.669180 | 0.07199565 | 1.351776 | 1.534328 | 4.120776 |
| 9.371943 | 18.037410 | 0.5772676 | 1.343091 | 1.073730 | 0.7853996 |
| 17.521350 | 11.119570 | 0.2410497 | 0.4620362 | 4.039876 | 3.732480 |
| 10.119520 | 17.787960 | 0.1191083 | 0.6453074 | 2.956790 | 2.806090 |
| 0.6522801 | 7.067789 | 1.824710 | 0.6382397 | 0.7485211 | 0.9193597 |
| 0.000000 | 5.357805 | 1.255946 | 0.694680 | 2.131141 | 0.2666985 |
| 23.453630 | 23.096980 | 0.3017777 | 2.594597 | 0.2739988 | 0.6914507 |
| 1.887341 | 21.665160 | 0.7274776 | 1.248168 | 0.4228202 | 0.2226693 |
| 7.042209 | 16.991860 | 0.3522972 | 2.568858 | 3.627325 | 0.2563953 |
| 3.552489 | 222.221000 | 0.3230295 | 1.438227 | 1.172399 | 1.033716 |
| 2.471314 | 17.239210 | 1.974943 | 2.933730 | 0.7378496 | 1.367028 |
| 17.317800 | 12.471390 | 1.018744 | 1.859691 | 0.8676586 | 11.890400 |
| 7.701060 | 13.891280 | 1.075203 | 1.010635 | 0.8757069 | 1.139843 |
| 2.633705 | 9.453052 | 2.053969 | 6.324391 | 2.481879 | 11.418870 |
| 42.057580 | 37.967040 | 1.692497 | 12.878750 | 0.01356691 | 42.056740 |
| 212.272900 | 6.036919 | 11.234310 | 5.020139 | 2.640738 | 0.5919393 |
| 111.935500 | 41.693700 | 30.614870 | 18.155990 | 0.8877779 | 11.394990 |
| 3.906460 | 18.016090 | 11.414200 | 8.375254 | 1.241985 | 0.2352567 |
| 2.945075 | 18.435550 | 2.433418 | 58.374270 | 0.7413745 | 0.8920985 |
| 41.144360 | 9.122541 | 1.517707 | 13.505980 | 0.9623976 | 3.586639 |
| Tumor | Normal | Tumor | Normal | Tumor | Normal |
| 1.137006 | 1.117729 | 5.226758 | 1.586779 | 2.183753 | 10.563870 |
| 1.591101 | 53.904430 | 1.544753 | 1.528191 | 0.9149762 | 3.176242 |
| 1.698718 | 0.2552336 | 3.166101 | 0.7720504 | 4.533696 | 10.578660 |
| 10.099890 | 11.278800 | 0.820326 | 1.607253 | 1.705343 | 2.540991 |
| 1.990862 | 17.503420 | 1.083652 | 0.3267922 | 6.289706 | 17.267850 |
| 6.845494 | 2.609828 | 1.526413 | 1.301650 | 7.292834 | 2.815618 |
| 3.889328 | 37.713360 | 1.480482 | 4.339987 | 4.087906 | 5.341807 |
| 3.014760 | 1.595839 | 1.199157 | 1.629646 | 2.441836 | 3.003414 |

2. The expression of SOX15 protein in liver cancer and normal tissues

GAPDH

**N T N T N T N T N T**


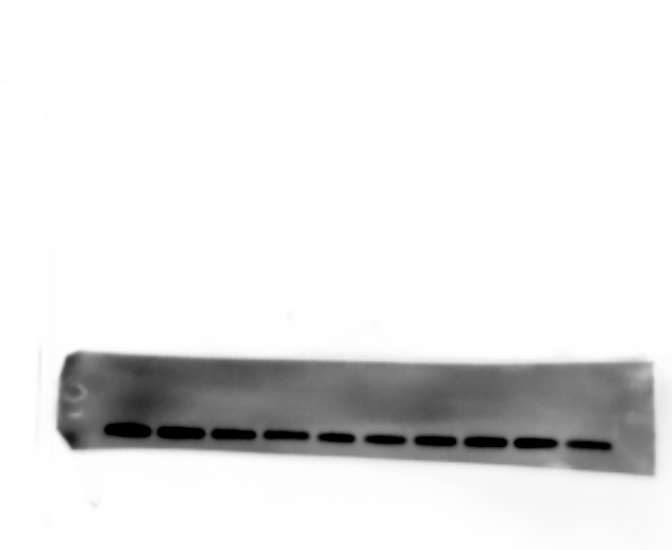


SOX15

**N T N T N T N T N T**


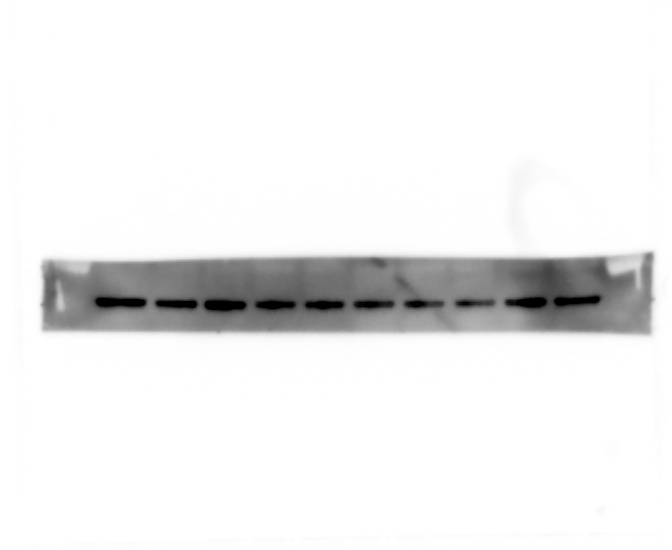


| Normal tissue | Tumor tissue |
| --- | --- |
| 1.060 | 0.60 |
| 1.090 | 0.51 |
| 0.970 | 0.42 |
| 0.910 | 0.53 |
| 0.920 | 0.83 |

3． IHC of SOX15 in liver cancer and normal tissues

Normal tissue (100X, 200X and 400X)


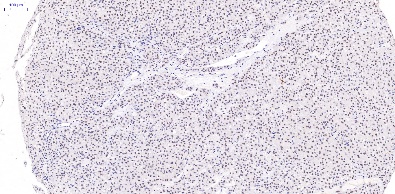


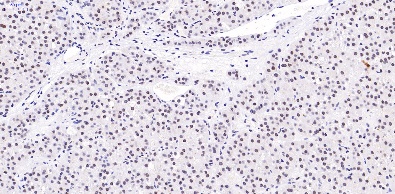


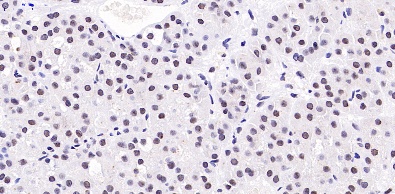


Tumor tissue (100X, 200X and 400X)


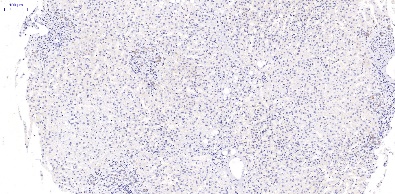


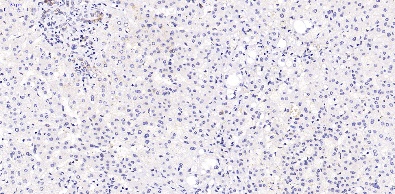


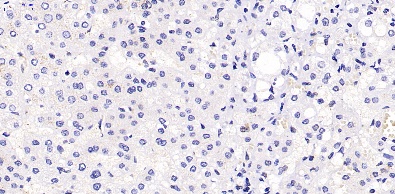


4. overall survival of liver cancer

| Survival time（months） | | SOX15 high | SOX15 low | Survival time（months） | SOX15 high | SOX15 low |
| --- | --- | --- | --- | --- | --- | --- |
| 19.6 | |  | 1 | 38.6 |  | 0 |
| 18.1 | |  | 1 | 6.9 |  | 1 |
| 45.1 | |  | 1 | 38.3 |  | 0 |
| 46.4 | |  | 1 | 38.1 |  | 0 |
| 48.3 | |  | 0 | 37.9 |  | 1 |
| 9.9 | |  | 1 | 37.7 |  | 0 |
| 6.2 | |  | 1 | 37.7 |  | 0 |
| 48.7 | |  | 1 | 37.6 |  | 1 |
| 12.3 | |  | 1 | 11.2 |  | 0 |
| 27.6 | |  | 1 | 16.1 |  | 0 |
| 44.6 | |  | 1 | 6.4 |  | 1 |
| 51.3 | |  | 0 | 36.4 |  | 1 |
| 44.2 | |  | 0 | 36.4 |  | 1 |
| 46.2 | |  | 1 | 16.6 |  | 1 |
| 34.2 | |  | 0 | 34.6 |  | 0 |
| 36.0 | |  | 0 | 32.4 |  | 1 |
| 13.2 | |  | 1 | 32.3 |  | 1 |
| 29.5 | |  | 0 | 32.2 |  | 1 |
| 46.6 | |  | 1 | 30.8 |  | 0 |
| 41.3 | |  | 1 | 9.9 |  | 1 |
| 73.5 | |  | 0 | 29.1 |  | 0 |
| 72.1 | |  | 1 | 28.0 |  | 1 |
| 15.6 | |  | 1 | 27.6 |  | 0 |
| 45.9 | |  | 0 | 26.0 |  | 1 |
| 26.7 | |  | 0 | 34.8 |  | 1 |
| 48.0 | |  | 1 | 13.6 |  | 1 |
| 49.4 | |  | 0 | 32.3 |  | 0 |
| 44.0 | |  | 0 | 32.2 |  | 1 |
| 43.7 | |  | 1 | 16.6 |  | 0 |
| 4.6 | |  | 1 | 26.0 |  | 1 |
| 66.3 | |  | 0 | 6.9 |  | 0 |
| 36.1 | |  | 1 | 38.3 |  | 1 |
| 40.2 | |  | 1 | 6.2 |  | 1 |
| 17.8 | |  | 1 | 43.2 |  | 1 |
| 39.7 | |  | 1 | 51.3 |  | 0 |
| 34.1 | |  | 1 | 19.6 |  | 1 |
| 19.5 | |  | 1 | 48.3 |  | 1 |
| 39.1 | |  | 1 | 27.6 |  | 1 |
| 1.8 | |  | 1 | 44.6 |  | 1 |
| 38.9 | |  | 0 | 44.2 |  | 1 |
| Survival time（months） | SOX15 high | | SOX15 low | Survival time（months） | SOX15 high | SOX15 low |
| 19.6 | |  | 0 | 19.6 |  | 0 |
| 18.1 | |  | 1 | 27.6 | 1 |  |
| 16.1 | |  | 1 | 27.2 | 0 |  |
| 36.4 | |  | 1 | 38.6 | 1 |  |
| 7.2 | | 1 |  | 45.1 | 0 |  |
| 48.9 | | 1 |  | 46.4 | 0 |  |
| 45.8 | | 0 |  | 48.3 | 1 |  |
| 40.3 | | 0 |  | 9.9 | 0 |  |
| 34.2 | | 0 |  | 48.7 | 0 |  |
| 40.3 | | 0 |  | 27.6 | 1 |  |
| 40.0 | | 0 |  | 44.6 | 0 |  |
| 40.0 | | 0 |  | 44.2 | 1 |  |
| 39.7 | | 0 |  | 18.1 | 0 |  |
| 39.4 | | 1 |  | 45.1 | 0 |  |
| 39.3 | | 0 |  | 46.4 | 1 |  |
| 38.8 | | 0 |  | 9.9 | 0 |  |
| 18.1 | | 0 |  | 6.2 | 0 |  |
| 15.0 | | 0 |  | 51.3 | 0 |  |
| 37.9 | | 0 |  | 36.4 | 1 |  |
| 36.7 | | 0 |  | 36.4 | 0 |  |
| 9.6 | | 0 |  | 16.6 | 0 |  |
| 34.8 | | 0 |  | 34.6 | 0 |  |
| 34.6 | | 0 |  | 34.2 | 0 |  |
| 34.2 | | 0 |  | 13.9 | 0 |  |
| 13.9 | | 0 |  | 32.4 | 0 |  |
| 13.6 | | 0 |  | 30.8 | 0 |  |
| 30.6 | | 1 |  | 30.6 | 0 |  |
| 30.4 | | 0 |  | 30.4 | 0 |  |
| 29.1 | | 0 |  | 9.9 | 0 |  |
| 27.2 | | 0 |  | 9.1 | 0 |  |
| 26.6 | | 0 |  | 29.1 | 0 |  |
| 34.6 | | 1 |  | 8.0 | 0 |  |

5. The expression of SOX15 mRNA in HCC cell lines

| LO2 | LM3 | 449 | HUH7 | SKHEP1 |
| --- | --- | --- | --- | --- |
| 1.000533 | 0.2862319 | 0.4621045 | 0.3952897 | 0.4938013 |
| 1.033201 | 0.2993282 | 0.4397703 | 0.4096143 | 0.4902114 |
| 0.9678658 | 0.2731355 | 0.4844387 | 0.3809652 | 0.4973913 |

6. The expression of SOX15 MSP in HCC cell lines

MSP LM3 HUH7 SK

NC 5-AZA NC 5-AZA NC 5-AZA


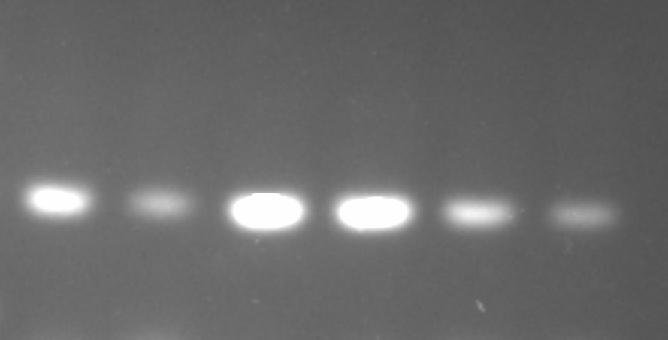


BSP LM3 HUH7 SK

NC 5-AZA NC 5-AZA NC 5-AZA


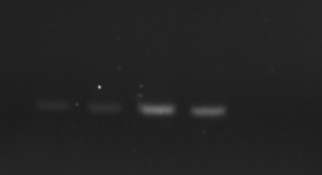


| LO2 | LM3 | 449 | HUH7 | SKHEP1 |
| --- | --- | --- | --- | --- |
| 0.99 | 10.2 | 2.1 | 9.7 | 10.1 |
| 1.01 | 11.5 | 2.3 | 10.5 | 9.7 |
| 1.02 | 9.8 | 1.8 | 8.9 | 9.9 |

7. The expression of SOX15 mRNA in HCC cell lines after 5-AZA administration

|  | NC | | | 5-AZA | | |
| --- | --- | --- | --- | --- | --- | --- |
| LM3 | 1.000006 | 1.003551 | 0.9964615 | 3.691642 | 4.036352 | 3.346931 |
| HUH7 | 1.001167 | 1.049487 | 0.9528466 | 3.541431 | 3.670623 | 3.412240 |
| SKHEP1 | 1.020799 | 0.8157855 | 1.225812 | 3.819207 | 3.779807 | 3.858608 |

8. The expression of SOX15 MSP in HCC cell lines 5-AZA administration

MSP LM3 HUH7 SK

NC 5-AZA NC 5-AZA NC 5-AZA


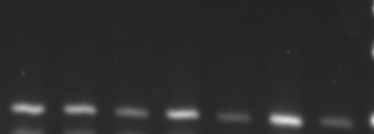


BSP LM3 HUH7 SK

NC 5-AZA NC 5-AZA NC 5-AZA


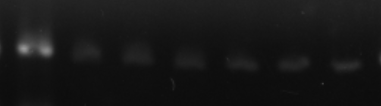


|  | NC | | | SOX15 | | |
| --- | --- | --- | --- | --- | --- | --- |
| LM3 | 1.00 | 1.01 | 0.99 | 0.05 | 0.08 | 0.07 |
| HUH7 | 1.10 | 0.93 | 0.97 | 0.16 | 0.13 | 0.14 |
| SKHEP1 | 1.06 | 0.99 | 0.95 | 0.07 | 0.09 | 0.06 |

9. SOX15 overexpression in HCC cell lines

SOX15 LM3 HUH7 SKHEP1

NC OE NC OE NC OE


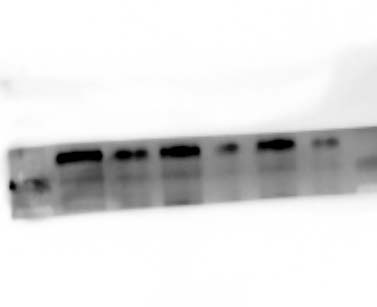


GAPDH LM3 HUH7 SKHEP1


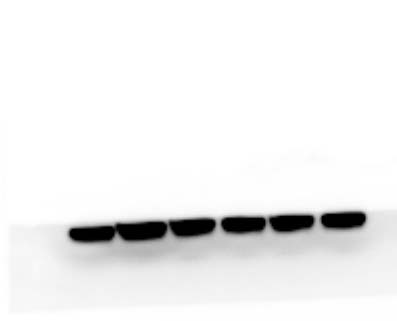


|  | NC | | | SOX15 | | |
| --- | --- | --- | --- | --- | --- | --- |
| LM3 | 0.95 | 1.09 | 0.98 | 8.30 | 7.90 | 8.70 |
| HUH7 | 0.99 | 0.98 | 1.03 | 10.30 | 11.10 | 9.70 |
| SKHEP1 | 1.05 | 0.98 | 0.97 | 5.60 | 6.10 | 6.70 |

10. The effects of SOX15 on cell proliferation by CCK8

| LM3 | NC | | | | | |
| --- | --- | --- | --- | --- | --- | --- |
| 6. | 0.928709 | 1.017341 | 1.036609 | 1.048170 | 0.9248555 | 1.044316 |
| 24. | 1.283237 | 1.418112 | 1.487476 | 1.421965 | 1.410405 | 1.379576 |
| 48. | 2.150289 | 2.250482 | 1.996146 | 1.973025 | 2.061657 | 1.984586 |
| 72. | 4.119461 | 3.807322 | 3.757226 | 3.934489 | 3.761079 | 3.845857 |
| 96. | 6.104046 | 5.660886 | 5.098266 | 5.333333 | 5.957611 | 5.922929 |
|  | SOX15 | | | | | |
| 6. | 0.9565217 | 0.9730849 | 0.9358178 | 1.072464 | 1.093168 | 0.9689441 |
| 24. | 0.9151139 | 1.051760 | 1.142857 | 1.225673 | 1.180124 | 1.039338 |
| 48. | 1.399586 | 1.540373 | 1.523810 | 1.449275 | 1.420290 | 1.407868 |
| 72. | 1.813665 | 1.855072 | 1.772257 | 1.850932 | 1.917184 | 1.896480 |
| 96. | 3.068323 | 2.923395 | 3.031056 | 3.358178 | 3.130435 | 2.691511 |

| HUH7 | NC | | | | | |
| --- | --- | --- | --- | --- | --- | --- |
| 6. | 0.9529412 | 1.001471 | 1.050000 | 1.014706 | 1.036765 | 0.9441177 |
| 24. | 1.244118 | 1.279412 | 1.544118 | 1.570588 | 1.601471 | 1.323529 |
| 48. | 2.673529 | 2.797059 | 2.492647 | 2.598529 | 1.839706 | 2.426471 |
| 72. | 3.228971 | 2.976618 | 3.469853 | 2.907794 | 3.068382 | 3.624706 |
| 96. | 4.345588 | 4.173530 | 3.794118 | 4.500000 | 4.354412 | 3.688235 |
|  | SOX15 | | | | | |
| 6. | 1.048163 | 1.033469 | 0.9991837 | 0.9306123 | 0.9795918 | 1.008980 |
| 24. | 1.062857 | 1.092245 | 1.425306 | 1.469388 | 1.400816 | 1.484082 |
| 48. | 1.621225 | 1.812245 | 1.743673 | 1.807347 | 1.689796 | 1.920000 |
| 72. | 1.833796 | 1.674612 | 1.954775 | 1.814694 | 1.865633 | 1.992980 |
| 96. | 1.920000 | 1.650612 | 1.880816 | 2.052245 | 2.027755 | 1.871020 |

| SK | NC | | | | | |
| --- | --- | --- | --- | --- | --- | --- |
| 6. | 0.9788785 | 0.9832485 | 0.9657685 | 1.013838 | 0.9613985 | 0.9788785 |
| 24. | 1.538238 | 1.555718 | 1.398398 | 1.494538 | 1.678077 | 1.538238 |
| 48. | 2.853606 | 2.980335 | 2.932266 | 2.460306 | 2.604516 | 2.853606 |
| 72. | 3.417335 | 3.618355 | 3.692644 | 3.517845 | 3.430445 | 3.417335 |
| 96. | 6.196651 | 6.262200 | 6.952660 | 6.576840 | 6.174800 | 6.196651 |
|  | SOX15 | | | | | |
| 6. | 0.9188812 | 0.9818183 | 1.044755 | 1.065734 | 0.9944057 | 1.006993 |
| 24. | 0.9104896 | 1.116084 | 1.120280 | 1.124476 | 1.225175 | 1.174825 |
| 48. | 1.615385 | 1.606993 | 1.623776 | 1.728672 | 1.409790 | 1.212588 |
| 72. | 2.039161 | 1.984616 | 2.064336 | 1.896504 | 1.913287 | 1.825175 |
| 96. | 3.096504 | 3.948252 | 3.990210 | 3.977623 | 4.111889 | 3.151049 |

10. The effects of SOX15 on cell colony formation

LM3 NC


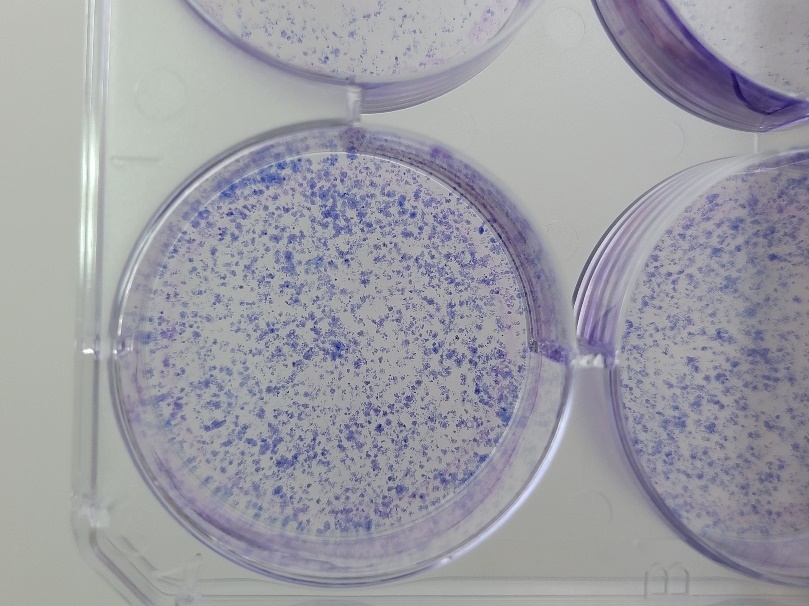


LM3 SOX15 OE


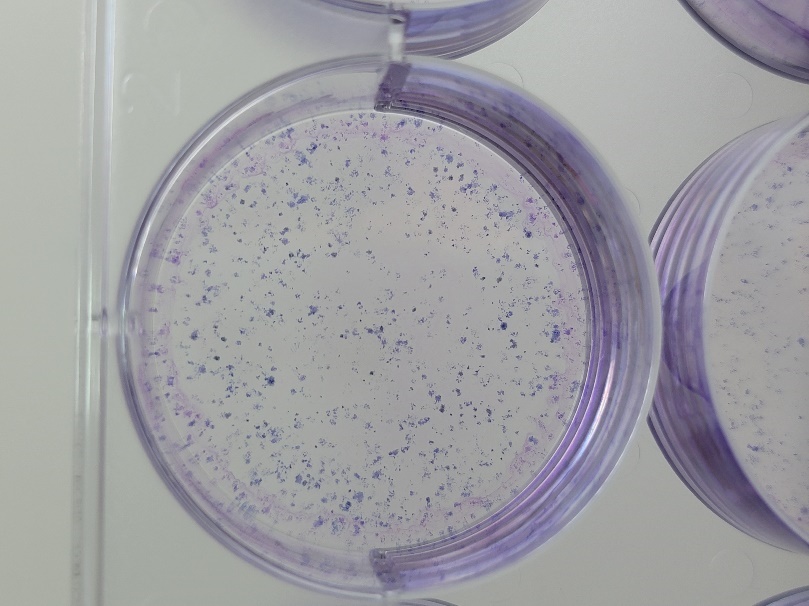


HUH7 NC


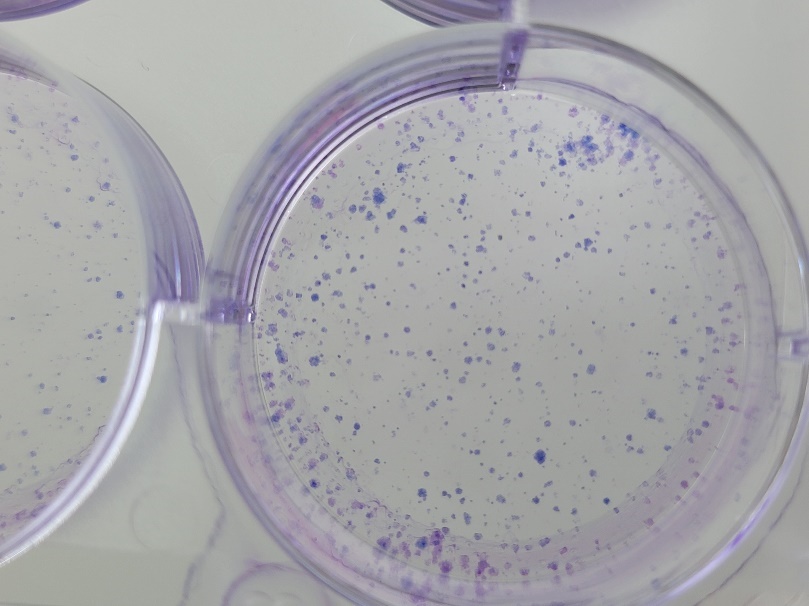


HUH7 SOX15 OE


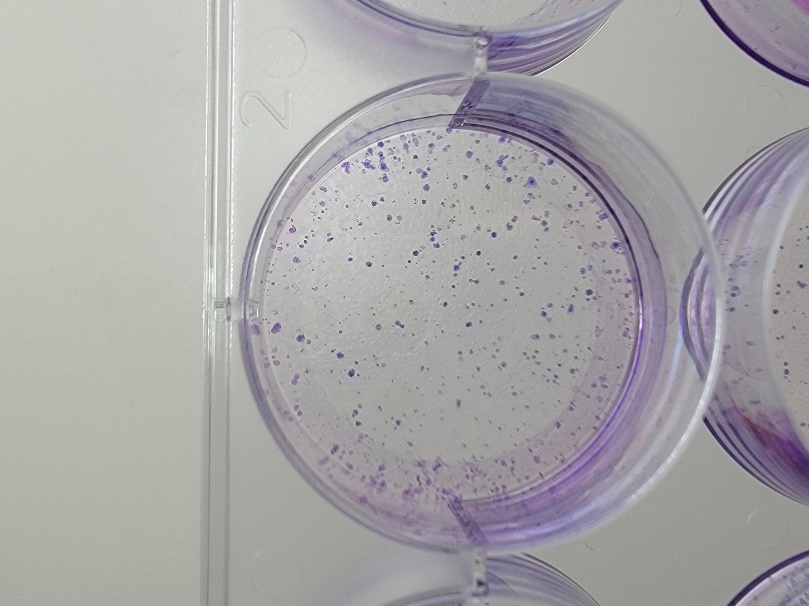


SKHEP1 NC


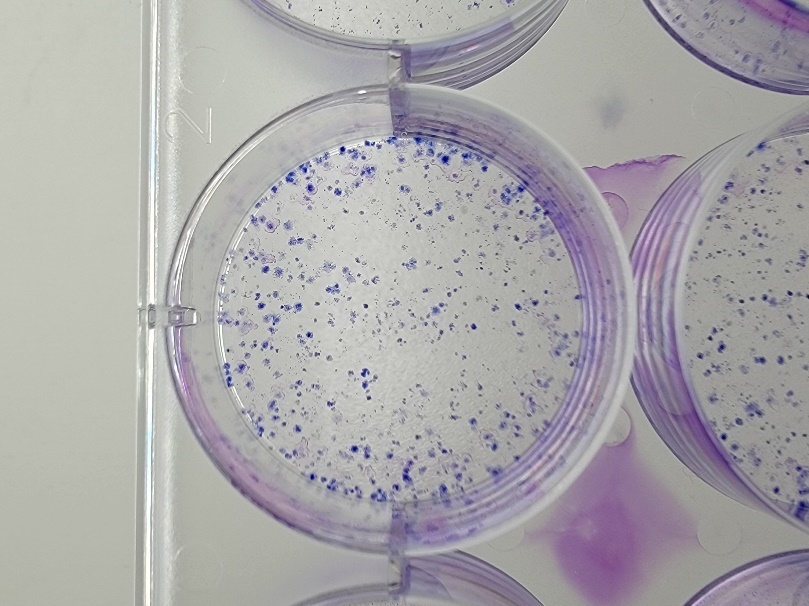


SKHEP1 SOX15 OE


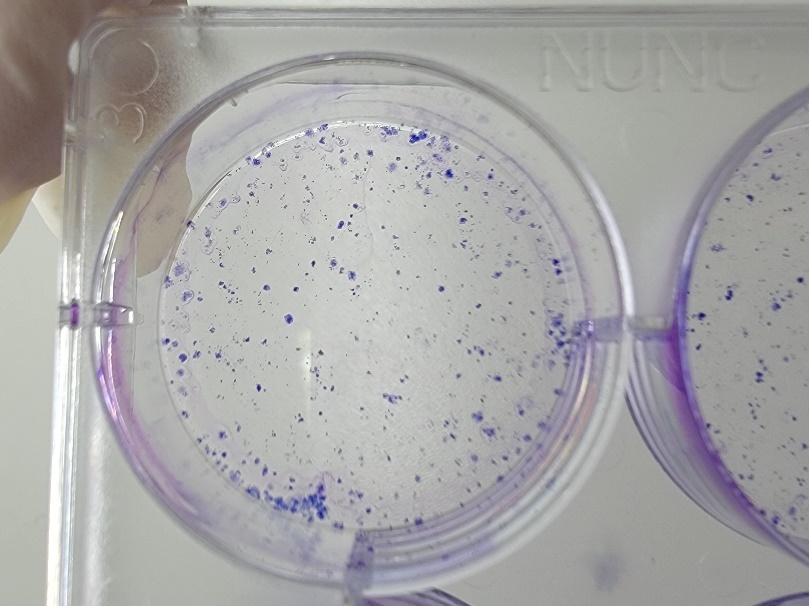


..

|  | NC | | | | | |
| --- | --- | --- | --- | --- | --- | --- |
| LM3 | 256. | 275. | 263. | 278. | 299. | 245. |
| HUH7 | 197. | 156. | 187. | 191. | 166. | 173. |
| SKHEP1 | 203. | 197. | 169. | 177. | 183. | 178. |
|  | SOX15 | | | | | |
| LM3 | 97. | 75. | 89. | 65. | 77. | 93. |
| HUH7 | 56. | 66. | 57. | 83. | 45. | 59. |
| SKHEP1 | 89. | 97. | 103. | 115. | 69. | 73. |

11 SOX15 overexpression in HCC tumors

SOX15 NC NC OE OE


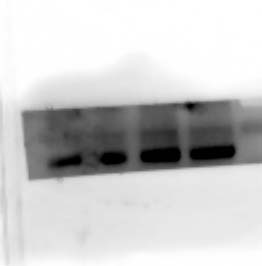


GAPDH NC NC OE OE


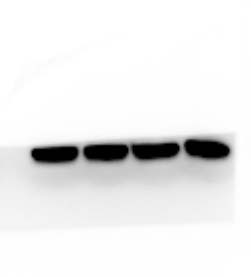


12. Tumor volume

|  | NC | | | | | |
| --- | --- | --- | --- | --- | --- | --- |
| 0. | 127.64300 | 75.271680 | 72.000000 | 149.251800 | 57.257280 | 55.86240 |
| 3. | 320.98620 | 171.181000 | 192.946400 | 266.271600 | 216.016100 | 115.87550 |
| 6. | 366.28520 | 471.955400 | 986.185500 | 591.591600 | 656.594400 | 150.21880 |
| 9. | 602.05680 | 531.841400 | 711.768600 | 1138.551000 | 1032.955000 | 369.30660 |
| 12. | 916.65120 | 1369.386000 | 716.187700 | 722.010400 | 1207.085000 | 271.24610 |
| 15. | 2310.56300 | 1903.247000 | 887.947400 | 1289.625000 | 994.768700 | 603.34950 |
|  | SOX15 | | | | | |
| 0. | 117.740800 | 58.471870 | 122.297300 | 69.53500 | 61.661250 | 130.186400 |
| 3. | 144.184300 | 189.100900 | 171.310900 | 137.31700 | 139.387100 | 152.742200 |
| 6. | 258.741000 | 547.611500 | 85.983660 | 281.10350 | 449.531700 | 76.196300 |
| 9. | 371.713400 | 431.101400 | 746.532000 | 272.80640 | 340.063900 | 103.761000 |
| 12. | 679.156900 | 530.226300 | 520.231500 | 381.25080 | 408.240000 | 432.961000 |
| 15. | 814.296000 | 757.950900 | 1483.729000 | 604.40950 | 849.799600 | 747.104400 |

13. 肿瘤图


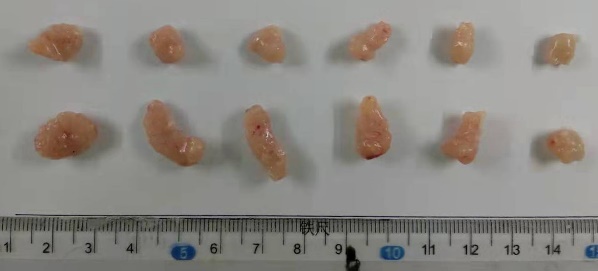


14. Tumor weight

| NC | SOX15 |
| --- | --- |
| 1.28 | 0.77 |
| 1.19 | 0.70 |
| 1.14 | 0.67 |
| 0.88 | 0.62 |
| 0.77 | 0.51 |
| 0.63 | 0.38 |

15. HE and Ki-67 staining

HE NC


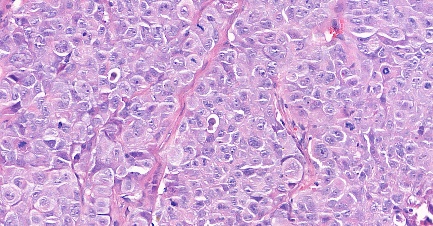


HE SOX15 OE


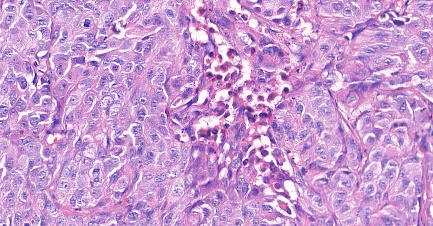


Ki-67 NC


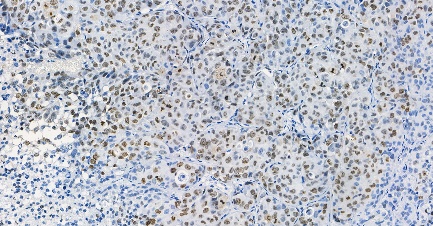


Ki-67 SOX15 OE


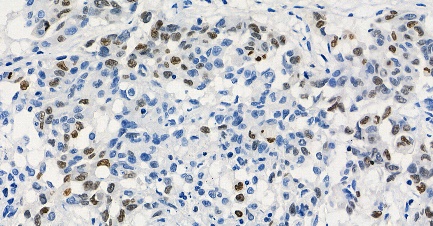


| NC | SOX15 |
| --- | --- |
| 79 | 55. |
| 82 | 51. |
| 71 | 49. |
| 69 | 66. |
| 74 | 43. |
| 77 | 47. |

16. western blot of β-catenin, CD44 and MET in HCC cell lines and mice tumor model

β-catenin LM3 HUH7 SKHEP1


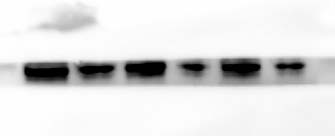


CD44 LM3 HUH7 SKHEP1


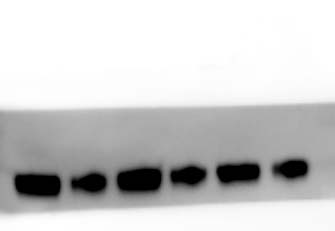


MET LM3 HUH7 SKHEP1


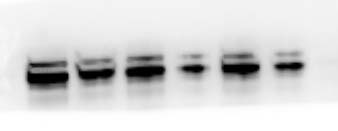


SOX15 LM3 HUH7 SKHEP1


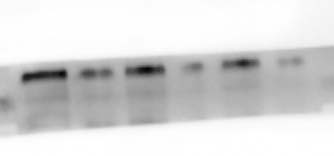


GAPDH LM3 HUH7 SKHEP1


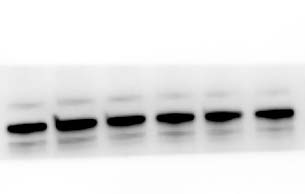


β-catenin NC NC OE OE


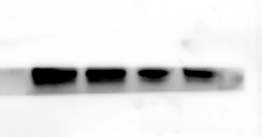


CD44 NC NC OE OE


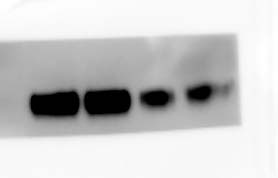


MET NC NC OE OE


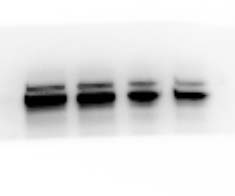


SOX15 NC NC OE OE


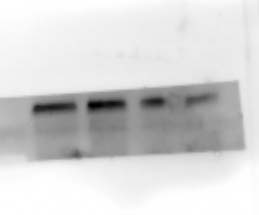


GAPDH NC NC OE OE


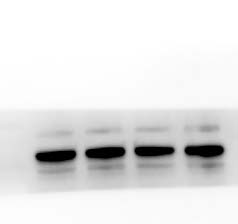


| LM3 | NC | | | SOX15 | | |
| --- | --- | --- | --- | --- | --- | --- |
| β-catenin. | 1.03 | 1.02 | 0.95 | 0.52 | 0.47 | 0.55 |
| CD44 | 0.98 | 0.97 | 1.03 | 0.65 | 0.59 | 0.67 |
| MET | 0.97 | 0.99 | 1.04 | 0.74 | 0.69 | 0.71 |

| HUH7 | NC | | | SOX15 | | |
| --- | --- | --- | --- | --- | --- | --- |
| 1.03 | 1.02 | 0.95 | 0.32 | 0.36 | 0.29 | 1.03 |
| 0.97 | 0.99 | 1.04 | 0.52 | 0.49 | 0.47 | 0.97 |
| 0.99 | 0.98 | 1.03 | 0.24 | 0.23 | 0.21 | 0.99 |

| SK | NC | | | SOX15 | | |
| --- | --- | --- | --- | --- | --- | --- |
| β-catenin. | 0.99 | 0.98 | 1.03 | 0.15 | 0.17 | 0.09 |
| CD44 | 0.96 | 1.02 | 1.02 | 0.54 | 0.45 | 0.49 |
| MET | 1.05 | 1.01 | 0.94 | 0.45 | 0.40 | 0.38 |

| Mice model | NC | | | SOX15 | | |
| --- | --- | --- | --- | --- | --- | --- |
| β-catenin. | 1.03 | 1.01 | 0.96 | 0.31 | 0.32 | 0.29 |
| CD44 | 1.06 | 1.03 | 0.91 | 0.23 | 0.26 | 0.31 |
| MET | 0.99 | 0.96 | 1.05 | 0.32 | 0.29 | 0.33 |
